# Total resection of sellar and suprasellar epidermoid cyst by using endoscopic endonasal approach: a case report

**DOI:** 10.1093/jscr/rjac203

**Published:** 2022-05-05

**Authors:** Muhammad Nour Alabdullah, Abdulrahman Awad, Ammar Abdullah, Hadi Alabdullah

**Affiliations:** Otorhinolaryngology Department, Al-Mowassat University Hospital, Damascus University, Damascus, Syrian Arab Republic; Otorhinolaryngology Department, Tishreen Military Hospital, Damascus, Syrian Arab Republic; Otorhinolaryngology Department, Tishreen Military Hospital, Damascus, Syrian Arab Republic; Faculty of Medicine, Hama University, Hama, Syrian Arab Republic

## Abstract

Epidermoid cysts (EC) are benign lesions resulting from incomplete separation of the neuroectoderm during embryonic development. The investigation of choice for EC is magnetic resonance imaging (MRI). Surgical resection is the treatment of choice. Full resection of EC including the cyst wall to prevent recurrence and malignant transformation should be considered when possible. Two main approaches were described in the literature and included craniotomy and endoscopic endonasal approach (EEA). Using of EEA to accomplish total resection could be challengeable. To best of our knowledge, only 6 manuscripts (with a total of eight patients) reported total resection of EC by using EEA. Our case should be the ninth such cases in the literature. In this paper, we reported a case of sellar and suprasellar epidermoid cyst which was resected completely by using EEA. We revealed the safety and efficacy of this approach in management of such cases.

## INTRODUCTION

Intracranial epidermoid cysts (EC) are rare, congenital and benign cystic lesions. They account for 0.04%–1% of intracranial tumors. The lesions often become symptomatic in third and fourth decade of life [1]. They result from incomplete separation of the neuroectoderm during embryonic development between the fifth and sixth weeks of gestation, resulting in trapping ectodermal remnants inside the cranium [2]. They are slowly growing leading to adherence to surrounding neurovascular structures. This makes the total resection of those lesions impossible in many cases [3]. Two main approaches were reported in the literature and included craniotomy and endoscopic endonasal approach (EEA). Total resection of EC by using EEA is challengeable. To best of our knowledge, only six manuscripts (with a total of eight patients) reported total resection of EC by using this approach. Our cases should be the ninth such cases in the literature. We reported this case to spotlight awareness about the safety and efficacy of EEA in treatment of sellar and parasellar EC with some literature review.

## CASE REPORT

**Figure 1:**
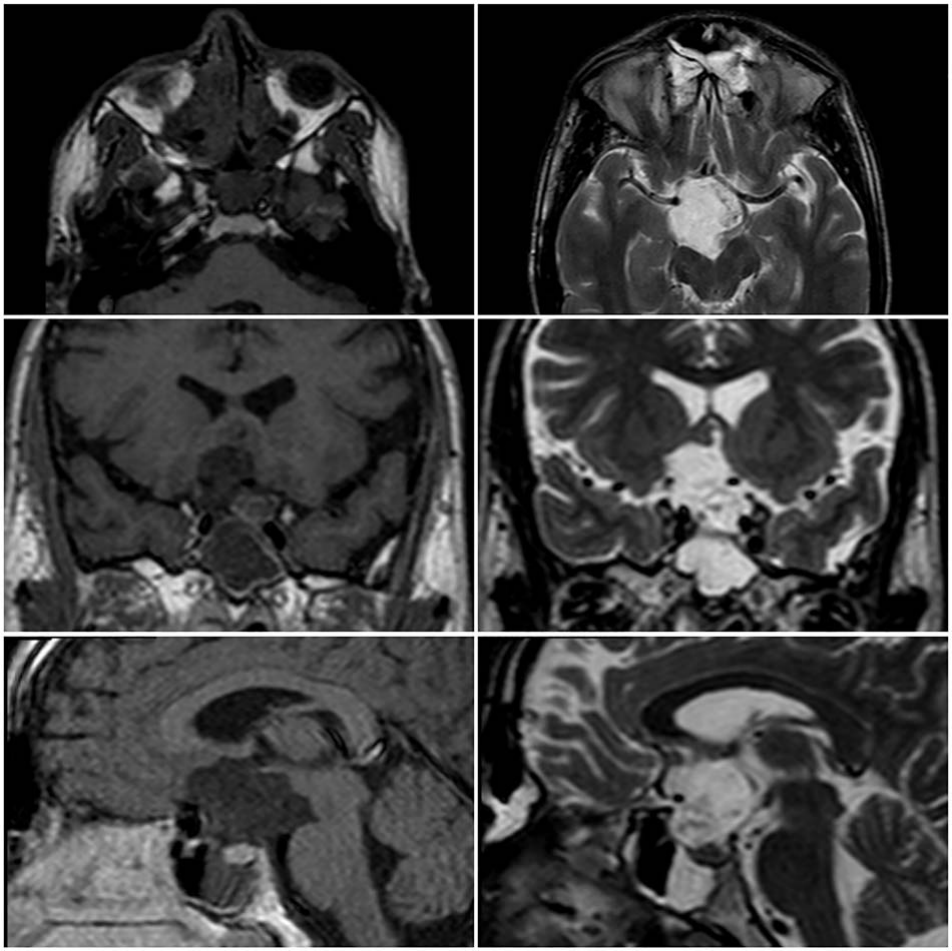
MRI in axial, coronal and sagittal views. The lesion appears with hyposignal in T1WI and hypersignal in T2WI.

**Figure 2:**
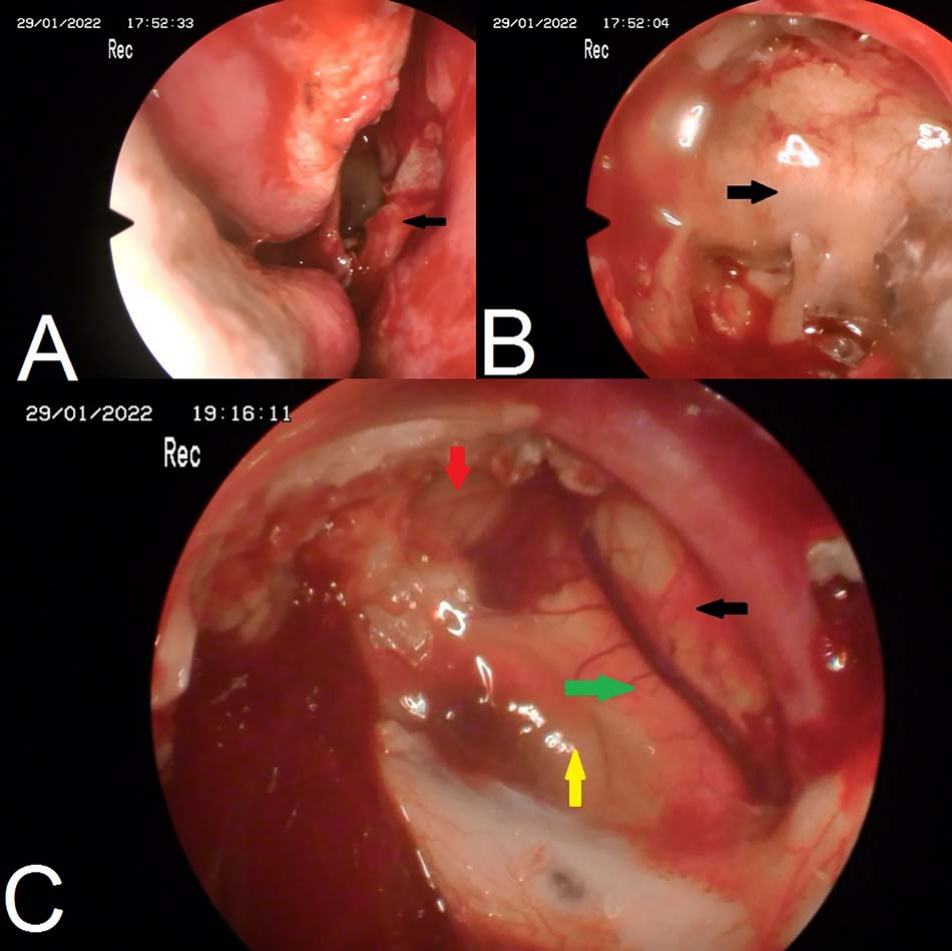
Endoscopic images. (**A**) nasoseptal flap; (**B**) posterior bony wall of sphenoid sinus; (**C**) complete resection of epidermoid cyst, green arrow: optic chiasm, yellow arrow: pituitary gland, red arrow: internal carotid artery, black arrow: anterior brain.

**Figure 3:**
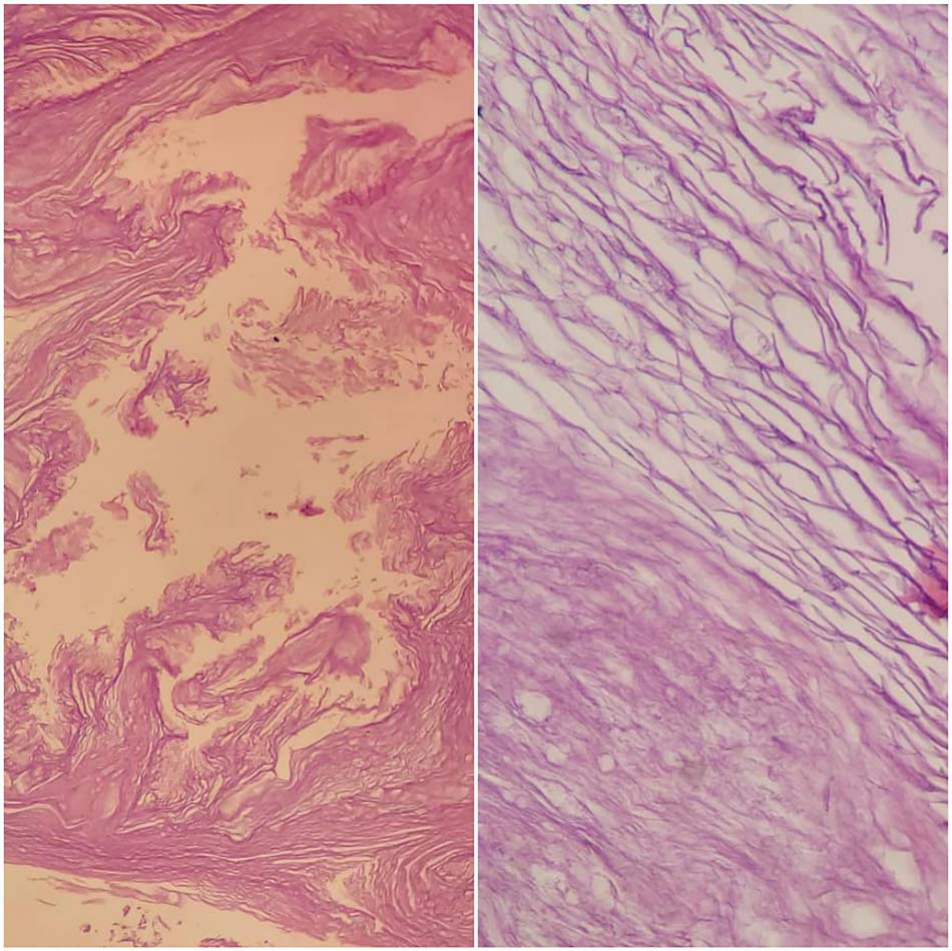
Histological examination of cyst contents. Haematoxilin-eosin stain. Keratin flakes.

A 22-year-old caucasian male was admitted to neurosurgery department with a history of chronic headache and 8-month history of bilateral visual acuity deterioration. Ophthalmologic evaluation revealed light perception on the right side and hand motion on the left side. Fundus examination showed bilateral temporal optic nerve pallor. Eye movement was normal. The patient had no focal neurological deficits. Laboratory tests were unremarkable. Magnetic resonance imaging (MRI) revealed a sellar and suprasellar well-defined cystic lesion with irregular margins, measures about 2.6 × 3.3 × 4 cm. The mass was hypointense in T1-weighted images (T1WI) and hyperintense in T2-weighted images (T2WI) with restriction in diffusion-weighted images (DWI). The mass was compressing the optic chiasm and surrounding the right optic nerve and pituitary stalk (Fig. 1). The patient underwent an endoscopic surgery and transnasal trans-sphenoidal approach was used to resect the cyst. A drilling of the sphenoid sinus septations was done. Using the Kerrison, the superior half of the posterior bony wall of the sphenoid sinus was removed to reach the sellar and suprasellar region. The dura was incised widely. We observed a sac within the sella compressing the pituitary gland. The sac was extended to the suprasellar region compressing the optic chiasm and reached to the floor of the frontal lobe anteriorly. The sac was opened and completely evacuated of cheesy materials. After evacuation of the cyst, we detached the epidermoid capsule from the pituitary gland and displaced the sac upward into the suprasellar region. A smooth dissection was applied to detach the capsule of epidermoid from the optic chiasm down to the posterior frontal lobe. We used a bipolar diathermy to coagulate small bleeding vessels. A 30° endoscope was used to confirm a complete resection of the sac (Fig. 2). An underlay graft from fascia lata of musculus quadriceps femoris was used to cover the defect of skull base and reinforced with fat tissue, nasoseptal flap and thrombin-soaked collagen sponges. Pathology revealed sheets of keratin indicating an EC (Fig. 3). Two days after surgery, the patient experienced sudden onset of high-grade fever and neck stiffness. The CSF opening pressure was normal and cultures for bacteria, mycobacteria and fungi were negative. The cell count and glucose and protein levels were normal. Based on the CSF findings, a diagnosis of aseptic meningitis was established. Conservative management was applied and the meningitis was resolved. Seven days after surgery, the patient showed partial recovery in visual acuity of the left eye with no improvement on the right side. On 6-month follow-up, the patient remained stable and no complication was developed. Brain MRI demonstrated no evidence of residual or recurrent lesion.

## DISCUSSION

An intracranial EC is a congenital lesion results from trapping ectodermal remnants inside the cranium during embryonic development [2]. The slow growth of those tumors delays the onset of symptoms until they become large and compress adjacent anatomical elements [4]. When the mass becomes contact with the optic chiasm, visual field defects become apparent [3]. The investigation of choice for EC is MRI. The treatment of choice is surgical resection [5]. Unfortunately, complete resection could be very challengeable due to severely adherent tumor capsule to the adjacent neural and vascular structures. The adhesion is thought to result from the inflammatory reaction between the contents of EC and the adjacent structures [3]. Complete resection should be considered when possible for two concepts. Firstly, incomplete resection may increase the rate of recurrence [6]. Secondly, some articles reported malignant transformation of EC [7]. Purely sellar and suprasellar ECs are very rare [3]. We reviewed the medical literature and found that only 29 cases have been reported in sellar and suprasellar region. Craniotomy approach was used in 12 cases out of 29, whereas EEA was used in the remaining 17 cases. Only eight cases out of the 17 cases reported full resection of EC (Table 1). Of the eight cases, two reported postoperative complications including reversible diabetes insipidus and hypocortisolism [3]. The use of EEA has several advantages compared with craniotomy. The most notable ones include avoidance of neurovascular retraction, optimal cosmetic results and shortening of surgical time and length of hospitalization. The EEA is ideal for resection of EC located in the ventral aspect of the skull base (cysts in the sellar or suprasellar, interpeduncular, prepontine or premedullary cisterns) particularly those measure <4 cm [8]**.** In our case, we successfully achieved complete resection by using endoscopic surgery and transnasal trans-sphenoidal approach. The patient had postoperative aseptic meningitis which was treated conservatively. It is important to keep in mind chemical meningitis due to intrathecal spillage of the cyst content during surgery [9]. In the conclusion, the reported cases showed the EEA as a safe and effective approach for most cases of sellar and parasellar ECs. Full resection should be achieved when possible. Longtime follow-up should be considered because this lesion may recur after a few years [9].

**Table 1:** previous reported cases of EC which have been total resected by using EEA

Author	Number of patients	Age/gender	Postoperative complications
Montaser *et al*. (2018)	1	49/F	None
McCormack *et al*. (2018)	1	36/F	Reversible diabetes insipidus
Huo *et al.* (2018)	1	42/F	Diabetes insipidus and hypocortisolism
Nakassa *et al*. (2018)	1	54/F	None
Forbes *et al*. (2018)	2	70/F 19/F	None
Vellutini *et al*. (2021)	2	44/M 70/M	Aseptic meningitis

F: female; M: male.
